# Adenomatoid odontogenic tumor

**DOI:** 10.1016/S1808-8694(15)31107-1

**Published:** 2015-10-19

**Authors:** Belmiro Cavalcanti do Egito Vasconcelos, Riedel Frota, Álvaro Bezerra Cardoso, Gabriela Granja Porto, Suzana Célia de Aguiar Soares Carneiro

**Affiliations:** 1 PhD, graduate programs coordinator - UPE; 2 M. Sc in Maxillo-Facial Surgery - University of Pernambuco, PhD. Student - Maxillo-Facial Surgery - University of Pernambuco; 3 Specialist in Maxillo-Facial Surgery and Trauma - University of Pernambuco. M. Sc. Student in Dentistry (oral diagnosis) - Universidade Federal da Paraíba; 4 Specialist in Maxillo-Facial Surgery and Trauma - University of Pernambuco. M. Sc. Student in Maxillo-Facial Surgery and Trauma - University of Pernambuco; 5 Specialist in Maxillo-Facial Surgery and Trauma - University of Pernambuco. M. Sc. Student in Maxillo-Facial Surgery and Trauma - University of Pernambuco

**Keywords:** maxilla/pathology, oral, pathology

## INTRODUCTION

The adenomatoid odontogenic tumor (AOT) is usually an asymptomatic slow growth lesion. When grown, one can palpate a hard and large lesion. It is common for the tumor to cause shifting of neighboring teeth because tumor expansion is more common than teeth root resorption. Radiographically, there is a unilocular mass involving an unerupted tooth, sometimes opaque in the center and sclerotic in the periphery. Considering it to be an encapsulated tumor, treatment of choice is enucleation^1^. This paper describes three cases of these tumors and their symptoms, their radiographic characteristics and anatomic findings.

## REPORT OF CASES

### Cases 1 and 2

**Figure 1:**
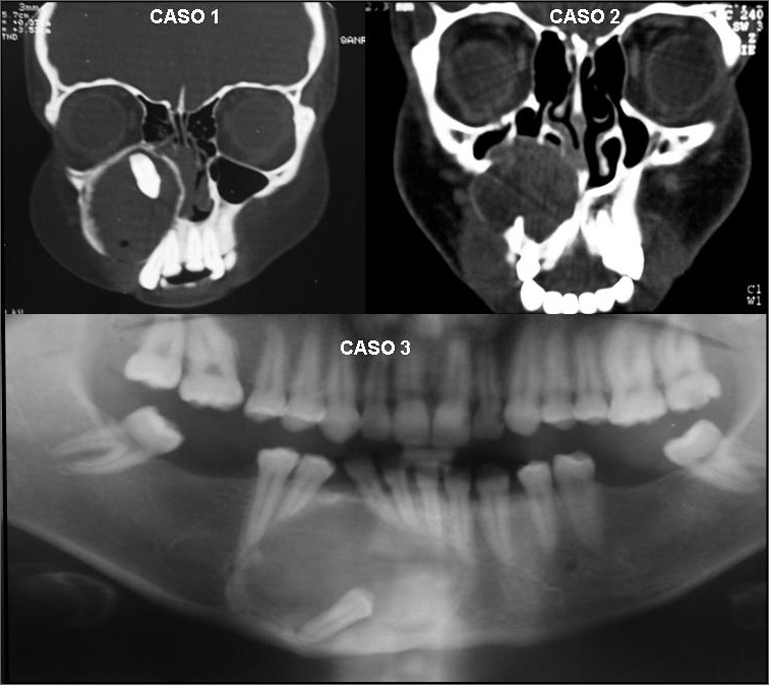
Radiographic aspects of cases 1, 2 and 3.

Both occurred on the second decade of life, the first in a girl and the second in a boy. The tumors evolved for about one year, they were asymptomatic and presented a hard and large mass in the paranasal region, obliterating the nasolabial groove and obstructing the nostril. Radiographic exams revealed an extensive unilocular image involving the maxillary sinus and the nasal cavity of the affected side (Figure 1). Treatment of choice was tumor enucleation under general anesthesia. The patients were followed up for one year after surgery, when there was bone regeneration.

### Case 3

This case refers to a 28 year-old patient, who had a tumor in the anterior portion of her mandible that had been growing for approximately 2 years (Figure 1). The tumor was also resected under general anesthesia and followed for one year.

## DISCUSSION

AOTs present a relative frequency of 2.2 to 7.1% among odontogenic tumors^2^. This type of tumor affects women more frequently (1.9:1) on their second decades of life. ^2^^,^^3^.

The maxilla location of this tumor is two fold more frequently than that in the mandible^4^, ^5^, ^6^. Progressive nasal obstruction is a common finding in lesions larger than 5.0cm located in the maxilla^5^.

AOTs have three variants (follicular, extra-follicular and peripheric), which make it very difficult to differentiate from other diseases (2). Differential diagnosis depends on the radiographic result, which may show a radiolucent area with or without radioopaqueness^5^. It is indispensable to make and incisional biopsy of the lesion for surgical planning purposes, as well as aspirating it before any procedure with radiolucent masses because they usually have a vascular origin.
